# COVID‐19 among adults living with HIV: correlates of mortality among public sector healthcare users in Western Cape, South Africa

**DOI:** 10.1002/jia2.26104

**Published:** 2023-06-20

**Authors:** Reshma Kassanjee, Mary‐Ann Davies, Olina Ngwenya, Richard Osei‐Yeboah, Theuns Jacobs, Erna Morden, Venessa Timmerman, Stefan Britz, Marc Mendelson, Jantjie Taljaard, Julien Riou, Andrew Boulle, Nicki Tiffin, Nesbert Zinyakatira

**Affiliations:** ^1^ School of Public Health University of Cape Town Cape Town South Africa; ^2^ Department of Health Western Cape Government Cape Town South Africa; ^3^ Wellcome Centre for Infectious Disease Research in Africa Institute of Infectious Diseases and Molecular Medicine University of Cape Town Cape Town South Africa; ^4^ Division of Computational Biology Integrative Biomedical Sciences Department Faculty of Health Sciences University of Cape Town Cape Town South Africa; ^5^ Department of Statistical Sciences University of Cape Town Cape Town South Africa; ^6^ Division of Infectious Diseases and HIV Medicine Department of Medicine Groote Schuur Hospital University of Cape Town Cape Town South Africa; ^7^ Division of Infectious Diseases Department of Medicine Tygerberg Hospital Stellenbosch University Cape Town South Africa; ^8^ Institute of Social and Preventive Medicine University of Bern Bern Switzerland; ^9^ South African National Bioinformatics Institute University of the Western Cape Cape Town South Africa

**Keywords:** HIV, SARS‐CoV‐2, COVID‐19, mortality, CD4 count, South Africa

## Abstract

**Introduction:**

While a large proportion of people with HIV (PWH) have experienced SARS‐CoV‐2 infections, there is uncertainty about the role of HIV disease severity on COVID‐19 outcomes, especially in lower‐income settings. We studied the association of mortality with characteristics of HIV severity and management, and vaccination, among adult PWH.

**Methods:**

We analysed observational cohort data on all PWH aged ≥15 years experiencing a diagnosed SARS‐CoV‐2 infection (until March 2022), who accessed public sector healthcare in the Western Cape province of South Africa. Logistic regression was used to study the association of mortality with evidence of antiretroviral therapy (ART) collection, time since first HIV evidence, CD4 cell count, viral load (among those with evidence of ART collection) and COVID‐19 vaccination, adjusting for demographic characteristics, comorbidities, admission pressure, location and time period.

**Results:**

Mortality occurred in 5.7% (95% CI: 5.3,6.0) of 17,831 first‐diagnosed infections. Higher mortality was associated with lower recent CD4, no evidence of ART collection, high or unknown recent viral load and recent first HIV evidence, differentially by age. Vaccination was protective. The burden of comorbidities was high, and tuberculosis (especially more recent episodes of tuberculosis), chronic kidney disease, diabetes and hypertension were associated with higher mortality, more strongly in younger adults.

**Conclusions:**

Mortality was strongly associated with suboptimal HIV control, and the prevalence of these risk factors increased in later COVID‐19 waves. It remains a public health priority to ensure PWH are on suppressive ART and vaccinated, and manage any disruptions in care that occurred during the pandemic. The diagnosis and management of comorbidities, including for tuberculosis, should be optimized.

## 1 INTRODUCTION

Three years have passed since the COVID‐19 pandemic began. A large proportion of people with HIV (PWH) have experienced SARS‐CoV‐2 infections, and this population appears to have a modestly increased risk of severe COVID‐19 outcomes [1, 2, 3, 4, 5, 6, 7]. However, since most published research has been from high‐income settings with almost all PWH on virally suppressive antiretroviral therapy (ART), there remains uncertainty about the role of HIV disease severity on COVID‐19 outcomes and the extent of mitigation with effective ART, especially in lower‐income settings [1]. The WHO Global Clinical Platform of COVID‐19 study showed that being on ART and/or virally suppressed was protective against poor COVID‐19 outcomes in hospitalized patients, but there are limited data on all COVID‐19 patients including those not admitted, and the effect of immunosuppression before the COVID‐19 episode [7, 8]. Furthermore, the effectiveness of vaccination in preventing severe COVID‐19 in PWH in this context remains unclear [9, 10].

A better understanding of the risk factors for severe COVID‐19 in PWH is vital to guide optimal use of anti‐SARS‐CoV‐2 therapies, especially in resource‐limited settings where access to these therapies remains extremely limited, but the majority of immunosuppressed and/or viraemic PWH live [11, 12]. Intermittent surges of COVID‐19 infections are likely to continue as the virus mutates, especially since most countries have relaxed COVID‐19 restrictions due to their unsustainable socio‐economic cost or most people are now being protected against severe disease by vaccination and/or prior infection. Severe COVID‐19 may also be more prolonged in PWH, facilitating the accumulation of mutations with a risk of variant evolution [13, 14].

We aimed to study the association between mortality and characteristics of HIV severity (CD4 cell count and HIV viral load [VL]) and management (ART collection and time since first HIV evidence) as well as COVID‐19 vaccination status among adult (aged ≥15 years) PWH with a diagnosed SARS‐CoV‐2 infection in the Western Cape (WC) province of South Africa (SA), adjusted for demographic characteristics, comorbidities, area‐specific COVID‐19 hospital admission pressure, location and time period.

## 2 METHODS

### 2.1 Study population and data sources

We studied adults (aged ≥15 years) with both HIV and SARS‐CoV‐2 infections who utilized public sector healthcare in the WC. The WC has about 5.5 million people aged ≥15 years with an estimated HIV prevalence of 14.2% among women and 7.7% among men [15, 16]. Approximately 75% of the population are dependent on public sector health services, but a greater proportion among PWH [17].

De‐identified data were extracted from the Western Cape Provincial Health Data Centre (WCPHDC), which uses a unique patient identifier to integrate patient‐level data from multiple information systems (administrative, laboratory, pharmacy and disease management) in all public sector health facilities in the province [17]. We included all adults who had: accessed healthcare in the 3 years preceding the start of the COVID‐19 epidemic in SA (1 March 2020), a laboratory‐confirmed positive SARS‐CoV‐2 PCR or antigen test in the public or private sector and evidence of HIV before or <3 weeks after the SARS‐CoV‐2 diagnosis. For most of the pandemic period, post‐mortem testing was required for all persons dying with symptoms consistent with COVID‐19 who had not been tested before death. We included all SARS‐CoV‐2 diagnoses (regardless of whether the SARS‐CoV‐2 infection resulted in hospitalization) until 10 March 2022 and data were extracted on 28 April 2022 to allow sufficient time to ascertain mortality. Due to the limited coverage and quality of diagnosis coding in our setting, HIV and other comorbidities were inferred by the WCPHDC from a range of sources, including laboratory tests, electronic disease management systems for HIV and tuberculosis, pharmacy dispensing data for specific conditions (e.g. combination ART for HIV; anti‐diabetic medication for diabetes) and ICD‐10 diagnostic codes [17]. Each inferred comorbidity was assigned an overall “first evidence date” when any source first indicated evidence of the condition. Among persons with any evidence of tuberculosis, a first evidence date for the *most recent episode* was calculated, where a single episode lasts for at most a year. The WCPHDC links data on people with diagnosed SARS‐CoV‐2 infection to the vital registration system on deaths weekly, using national identification numbers, and reviews all COVID‐19‐related deaths daily.

We obtained vaccination data by linking the South African national identifier to the Electronic Vaccine Data System which records all COVID‐19 vaccines administered nationally. The following vaccines were available as a primary series to the general population from 17 May 2021, starting with those aged ≥60 years, with progressive access for younger age groups: Janssen/Johnson & Johnson (Ad26.COV2.S) (initially a single dose for those aged ≥18 years, with second doses available from January 2022) and two doses of Pfizer‐BioNTech (BNT162b2). By 20 October 2021, everyone aged ≥12 years was eligible for vaccination. Immunocompromised individuals including PWH with CD4<200 cells/μl were eligible for an additional homologous vaccine dose (i.e. a second Ad26.COV2.S or third BNT162b2), while further booster doses were available to all aged ≥18 years 180 days after the previous dose from January 2022.

The study was approved by the University of Cape Town Health Research Ethics committee and the Western Cape Government Provincial Department of Health. The individual informed consent requirement was waived for this secondary analysis of de‐identified data.

### 2.2 Definitions of the mortality outcome, HIV characteristics and covariates

An outcome of death was recorded if death occurred within 14 days before to 28 days after the date of SARS‐CoV‐2 diagnosis (i.e. reporting of the result to the WCPHDC) or within 14 days after discharge from a hospitalization that started within 21 days of the SARS‐CoV‐2 diagnosis, and there was no clear non‐COVID‐19 cause of death recorded.

For CD4 cell count, we extracted the most recent CD4 within the previous 18 months up to 2 weeks before the SARS‐CoV‐2 diagnosis, and, for secondary analyses, the CD4 nadir and CD4 at ART initiation (closest to, and within 6 months before and 2 weeks after, ART initiation). For ART, we considered whether there was any evidence of ART collection >3 days before SARS‐CoV‐2 diagnosis. For VL, among those with ART evidence, we used the most recent measurement obtained within 24 months before to 2 weeks after the SARS‐CoV‐2 diagnoses. CD4 and VL were coded as “Unknown” when there was no recorded measurement. Time since the first HIV evidence was also considered.

Demographic covariates included were sex and age. We included indicators of the chronic conditions diabetes, hypertension, chronic kidney disease (CKD) and chronic obstructive pulmonary disease (COPD). PWH were considered to have these conditions during the SARS‐CoV‐2 episode if the first evidence date was before or <3 months after SARS‐CoV‐2 diagnosis, except for COPD where the first evidence needed to occur >2 weeks before the SARS‐CoV‐2 diagnosis (later evidence may be a consequence of COVID‐19 or incorrectly inferred from medication prescribed during the COVID‐19 episode). Experience of tuberculosis was categorized as any of: absence of evidence of tuberculosis, or, among those with evidence, time since the most *recent* start of a tuberculosis episode (≥ 1 year, <1 year or “ongoing”—where ongoing consists of episodes beginning <2 months before and ≤1 month after the SARS‐CoV‐2 diagnosis). An indicator of current pregnancy was also included.

Vaccination status at the time of SARS‐CoV‐2 diagnosis was assigned the highest level of protection from among six categories, based on time since and number of vaccines received: no vaccine received, <28 days since a first dose of either vaccine type (“early” vaccination), ≥28 days since a first vaccine dose (either Janssen or Pfizer–BioNTech, separately) or ≥14 days since a second dose (by type). We sometimes combined categories indicating a completed primary series (two Pfizer doses or ≥1 Janssen dose). No persons had third vaccines during our analysis period (defined below).

Variation by location and time were described by district, where the province is sub‐divided into six districts, and wave period, where four distinct waves coincided with periods of different variants being dominant in the WC, namely the ancestral, Beta, Delta and Omicron BA.1/BA.2 variants, respectively (wave 1: 2 May−6 Aug 2020; wave 2: 13 Nov 2020−1 Feb 2021; wave 3: 3 June−19 Sep 2021; wave 4: 28 Nov 2021−24 Feb 2022; inter‐wave periods: remaining time; where waves were defined as the periods when the 7‐day moving average of new COVID‐19 diagnoses was ≥ 60/million population). Additionally, we constructed a time‐specific proxy of the availability of healthcare: per district, admission pressure was the week's total number of COVID‐19 admissions relative to the district's maximum weekly COVID‐19 admissions (as a percentage).

### 2.3 Statistical analysis

Data management and analyses were conducted in SQL server Management Studio (version 8), STATA (version 15.1) and R (version 4.05).

In the primary analysis, we included infections until the end of wave 3 because of concerns that the SARS‐CoV‐2 diagnosis may be co‐incidental in a sizable proportion of deaths in those with SARS‐CoV‐2 infection in the fourth (Omicron BA.1/BA.2) wave when the prevalence of mild/asymptomatic COVID‐19 was high and there was extensive testing of all hospitalized patients [18]. Because reinfection risk was minimal until the end of the (third) Delta wave [3], only the first documented infections per person were included in the regression analyses described below, also to ensure the statistical independence of data points.

We used multivariable logistic regression to assess the association between mortality and HIV characteristics (recent CD4 cell count, recent VL, ART collection and time since first HIV evidence) as well as vaccination, adjusted for the person's characteristics (sex, age, diabetes, hypertension, CKD, COPD, tuberculosis and time since tuberculosis, and pregnancy) and time and location (district, wave period and admission pressure). All continuous variables were categorized a priori to ease interpretation. We included all terms as main effects and used likelihood ratio tests to obtain covariate‐level *p*‐values. To improve model‐fit (i.e. increase agreement of observed to model‐expected proportions experiencing death), we: fitted separate models to each of two age groups (15−39 years vs. ≥ 40 years old, based on dividing persons by the median age approximately) to account for potentially varying relationships of covariates with mortality by age; and included as an additional covariate an indicator of whether there were >2 co‐existing conditions (chronic conditions and ongoing/previous tuberculosis).

In secondary analyses (Supplementary Content), alternative models used either CD4 nadir or CD4 at ART initiation, instead of the most recent CD4; or analysed all first‐diagnosed cases until 10 March 2022.

## 3 RESULTS

**Figure 1:**
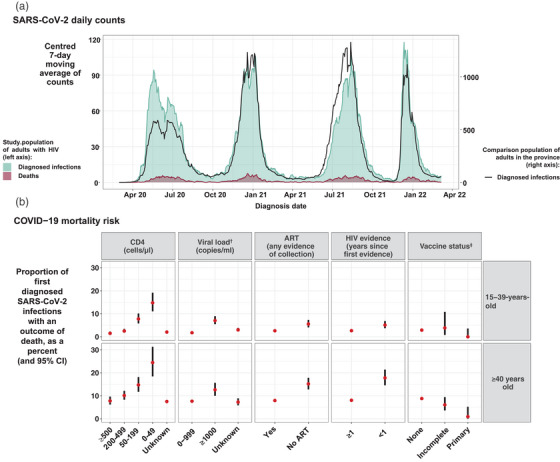
Description of SARS‐CoV‐2 infections and deaths among adults with HIV accessing public sector healthcare in the Western Cape Province: (a) counts of diagnosed SARS‐CoV‐2 infections and deaths over time, compared to diagnosed infections in the general population; and (b) the risk of mortality by HIV characteristics and vaccination in people who are experiencing a first diagnosed infection up to 10 March 2022, stratified by age. ^†^ Among those with evidence of ART. ^‡^ For descriptive purposes, ≥28 days since one Janssen vaccine, possibly with a second vaccine too, and ≥14 days since a second Pfizer vaccine, were combined into the “primary” series, and “early vaccination” (<28 days since a vaccine) or one Pfizer vaccine combined into “incomplete.” Abbreviations: ART, antiretroviral therapy; CI, confidence interval.

About 4.2% of the nearly 600,000 adults with HIV in the WC who accessed public health services in the 3 years before March 2020 were diagnosed with SARS‐CoV‐2 infection by 10 March 2022. While the general population experienced much higher infection peaks during subsequent COVID‐19 waves compared to the first, waves 1 to 4 in adult PWH had more consistent peaks (Figure 1a), with a much steeper rise in infections during the first wave, as well as the most infections that occurred in PWH being registered during this wave (see SDC A for additional descriptive statistics). The risk of mortality differed by HIV characteristics and vaccination status (Figure 1b).

We recorded 18,120 SARS‐CoV‐2 infections in PWH during the first three COVID‐19 waves, of which 22.5% were accompanied by a hospitalization that started within 21 days of the infection. Among all SARS‐CoV‐2 infections, 5.7% (1027) were associated with death (Table 1). The median age of SARS‐CoV‐2‐infected PWH was 39 years (interquartile range IQR: 32,47) and nearly three‐quarters (71.0%) were female. Half (51.4%) of infections were in PWH with ≥1 other chronic condition or ongoing/previous tuberculosis, and 15.5% of persons had multiple such conditions. Tuberculosis (ongoing/previous) was experienced by three in 10 (29.9%) persons, and hypertension was the most prevalent chronic condition (20.5%), followed by diabetes and COPD (9.7% and 7.2%). Chronic conditions were up to seven times more prevalent in those aged ≥40 years versus younger.

**Table 1:** Outcomes, demographic characteristics and co‐existing conditions for SARS‐CoV‐2 infections among adults with HIV in the Western Cape, stratified by age group (percentages and counts).

	Cases until end of wave 3			All cases (including wave 4)
Characteristic/outcome	Ages 15–39 years *n* = 9321	Ages ≥40 years *n* = 8799	All ages *n* = 18,120	All ages *n* = 22,057
**Death**	2.9 (270)	8.6 (757)	5.7 (1027)	5.6 (1233)
**Sex**				
Female	77.8 (7249)	63.8 (5610)	71.0 (12,859)	70.5 (15,561)
Male	22.2 (2072)	36.2 (3189)	29.0 (5261)	29.5 (6496)
**Age (years)**				
15–24	9.6 (895)	−	4.9 (895)	5.0 (1106)
25–29	21.6 (2011)	−	11.1 (2011)	11.4 (2517)
30–34	32.8 (3057)	−	16.9 (3057)	17.4 (3841)
35–39	36.0 (3358)	−	18.5 (3358)	18.7 (4130)
40–49	−	60.1 (5286)	29.2 (5286)	28.6 (6308)
50–59	−	29.4 (2590)	14.3 (2590)	13.9 (3057)
60–69	−	8.2 (722)	4.0 (722)	3.9 (864)
≥ 70	−	2.3 (201)	1.1 (201)	1.1 (234)
**Hypertension**	8.7 (809)	33.1 (2914)	20.5 (3723)	19.8 (4360)
**Diabetes**	4.0 (369)	15.8 (1391)	9.7 (1760)	9.1 (1999)
**Chronic obstructive pulmonary disease**	4.1 (379)	10.4 (917)	7.2 (1296)	7.8 (1714)
**Chronic kidney disease**	0.9 (86)	6.6 (584)	3.7 (670)	3.6 (784)
**Tuberculosis** ^a^ **(years since)**				
Absent	73.3 (6830)	66.7 (5868)	70.1 (12,698)	68.3 (15,068)
≥ 1	18.0 (1674)	26.4 (2323)	22.1 (3997)	22.3 (4911)
< 1	3.6 (338)	2.7 (238)	3.2 (576)	3.7 (806)
Ongoing	5.1 (479)	4.2 (370)	4.7 (849)	5.8 (1272)
**Pregnancy (in women aged 15–49 years)**	10.8 (784)	1.4 (50)	7.7 (834)	7.8 (1028)
**Number of previous SARS‐CoV‐2 diagnoses**				
0	98.1 (9146)	98.6 (8675)	98.3 (17,821)	96.6 (21,316)
1	1.9 (173)	1.4 (121)	1.6 (294)	3.3 (722)
2	0.0 (2)	0.0 (3)	0.0 (5)	0.1 (19)
**Number of conditions** ^b^				
0	62.4 (5814)	36.0 (3169)	49.6 (8983)	48.7 (10,746)
1	31.7 (2957)	38.3 (3368)	34.9 (6325)	35.7 (7872)
2	5.1 (476)	17.9 (1572)	11.3 (2048)	11.5 (2533)
≥3	0.8 (74)	7.8 (690)	4.2 (764)	4.1 (906)

^a^ Time since the most recent evidence of a start of an episode; with episodes beginning after 2 months before the SARS‐CoV‐2 diagnosis considered as ongoing.

^b^ Hypertension, diabetes, chronic obstructive pulmonary disease, chronic kidney disease and tuberculosis (at any time).

During the first three waves, very few of the people experiencing an infection (1.3%) had received their primary vaccination series (Table 2). For the HIV characteristics, a recent CD4 cell count was available for only 36.6% of persons, with a median of 446 (IQR: 274,648) cells/μl. Nearly, one in 10 (9.1%) infected persons had never collected ART. Among those with evidence of ART, in every 10 persons, one (9.1%) had a most recent VL ≥1000 copies/ml, while two (19.0%) had no recent measure. The median time since first HIV evidence was 6.3 years (IQR: 3.4,10.2).

**Table 2:** HIV characteristics and vaccination status for SARS‐CoV‐2 infections among adults with HIV in the Western Cape, stratified by age group (percentages and counts).

	Cases until end of wave 3			All cases (including wave 4)
Characteristic	Ages 15–39 years *n* = 9321	Ages ≥40 years *n* = 8799	All ages *n* = 18,120	All ages *n* = 22,057
**CD4 count (cells/μl)**				
≥ 500	14.0 (1304)	11.3 (991)	12.7 (2295)	12.6 (2775)
200–499	15.7 (1465)	11.5 (1014)	13.7 (2479)	13.5 (2986)
50–199	7.0 (648)	5.8 (511)	6.4 (1159)	7.1 (1576)
0–49	3.6 (333)	2.2 (191)	2.9 (524)	3.5 (773)
Unknown	59.8 (5571)	69.2 (6092)	64.4 (11,663)	63.2 (13,947)
**Viral load** ^a^ **(copies/ml)**				
0–999	66.4 (5642)	77.7 (6196)	71.9 (11,838)	69.6 (13,913)
≥ 1000	10.8 (915)	7.3 (585)	9.1 (1500)	10.0 (2003)
Unknown	22.8 (1941)	15.0 (1194)	19.0 (3135)	20.4 (4068)
**ART (years since most recent collection)**				
≥ 2	44.2 (4116)	46.5 (4092)	45.3 (8208)	46.0 (10,146)
< 2	47.0 (4382)	44.1 (3883)	45.6 (8265)	44.6 (9838)
No ART	8.8 (823)	9.4 (824)	9.1 (1647)	9.4 (2073)
**HIV evidence (years since first)**				
<1	9.0 (838)	5.9 (519)	7.5 (1357)	7.7 (1706)
1–5	39.2 (3653)	22.8 (2002)	31.2 (5655)	30.9 (6826)
5–10	36.5 (3404)	33.9 (2986)	35.3 (6390)	35.3 (7781)
≥ 10	15.3 (1426)	37.4 (3292)	26.0 (4718)	26.0 (5744)
**Vaccination status** ^b^				
None	97.9 (9126)	95.2 (8378)	96.6 (17,504)	89.6 (19,761)
Early	0.8 (74)	2.6 (126)	1.6 (300)	1.7 (379)
Janssen—one vaccine	1.2 (111)	1.1 (98)	1.2 (209)	3.7 (819)
Pfizer—one vaccine	0.1 (9)	0.9 (82)	0.5 (91)	1.3 (291)
Janssen—two vaccines	0.0 (0)	0.0 (0)	0.0 (0)	0.6 (137)
Pfizer—two vaccines	0.0 (1)	0.2 (15)	0.1 (16)	3.0 (670)

Abbreviation: ART, antiretroviral therapy.

^a^ Among those with evidence of ART at any time.

^b^ The highest level of protection from among six categories: no vaccine received (“None”); <28 days since a first vaccine dose (“Early”); ≥28 days since a first dose of a vaccine, by type (“one vaccine”); or ≥14 days since a second dose, by type (“two vaccines”).

By wave, over time, there was an increasingly higher prevalence of diagnosed infections with low recent CD4 cell counts <200 cells/μL, unsuppressed recent VL, no previous ART, ongoing tuberculosis or COPD (Tables 3 and 4). Consistent with vaccines becoming available to all ages shortly before wave 4, the percent of our population vaccinated increased from wave 3 (4.1%) to 4 (38.9%).

**Table 3:** Outcomes, demographic characteristics and co‐existing conditions for SARS‐CoV‐2 infections among adults with HIV in the Western Cape, stratified by wave (percentages and counts).

Characteristic/outcome	Wave 1 *n* = 5783	Wave 2 *n* = 5001	Wave 3 *n* = 5478	Wave 4 *n* = 3430	Inter‐wave *n* = 2361
**Death**	5.6 (323)	5.6 (281)	5.9 (325)	4.9 (167)	5.8 (137)
**Sex**					
Female	75.2 (4351)	70.3 (3515)	67.2 (3681)	68.9 (2364)	69.8 (1650)
Male	24.8 (1432)	29.7 (1486)	32.8 (1797)	31.1 (1066)	30.2 (715)
**Age (years)**					
15–24	4.8 (279)	4.1 (204)	5.1 (277)	4.7 (161)	7.8 (185)
25–29	11.3 (655)	10.9 (544)	10.3 (566)	12.7 (435)	13.4 (317)
30–34	17.5 (1012)	16.6 (830)	15.9 (872)	20.3 (695)	18.3 (432)
35–39	19.1 (1106)	17.8 (891)	18.5 (1016)	19.5 (669)	18.9 (448)
40–49	28.7 (1657)	29.7 (1486)	30.2 (1653)	26.1 (894)	26.1 (618)
50–59	13.7 (792)	14.8 (742)	15.0 (822)	12.2 (419)	11.9 (282)
60–69	3.9 (225)	4.7 (233)	4.0 (217)	3.7 (127)	2.6 (62)
**≥**70	1.0 (57)	1.4 (71)	1.0 (55)	0.9 (30)	0.9 (21)
**Hypertension**	21.2 (1226)	21.2 (1062)	20.0 (1096)	16.3 (559)	17.6 (417)
**Diabetes**	11.8 (684)	10.1 (506)	7.6 (418)	6.2 (212)	7.6 (179)
**Chronic obstructive pulmonary disease**	5.9 (343)	7.7 (384)	8.1 (442)	10.6 (365)	7.6 (180)
**Chronic kidney disease**	4.0 (233)	3.7 (183)	3.4 (187)	2.8 (95)	3.6 (86)
**Tuberculosis** ^a^ **(years since)**					
Absent	72.2 (4177)	71.7 (3586)	67.5 (3695)	60.8 (2087)	64.4 (1523)
≥ 1	21.5 (1242)	21.3 (1064)	23.8 (1303)	23.3 (800)	21.2 (502)
< 1	3.0 (174)	2.7 (136)	3.4 (187)	5.7 (194)	4.9 (115)
Ongoing	3.3 (190)	4.3 (215)	5.3 (293)	10.2 (349)	9.5 (225)
**Pregnancy (in women aged 15–49 years)**	7.1 (263)	7.5 (216)	7.0 (215)	8.1 (165)	11.7 (169)
**Number of previous SARS‐CoV‐2 diagnoses**					
0	100.0 (5783)	98.4 (4920)	96.9 (5308)	87.8 (3011)	97.0 (2294)
1	0.0 (0)	1.6 (81)	3.0 (166)	11.8 (406)	2.9 (69)
2	0.0 (0)	0.0 (0)	0.1 (4)	0.4 (13)	0.1 (2)
**Number of conditions** ^b^					
0	50.9% (2941)	50.2% (2509)	47.9% (2626)	45.1% (1546)	47.5% (1124)
1	33.1% (1917)	33.9% (1693)	37.0% (2028)	39.1% (1340)	37.8% (894)
2	11.3% (655)	11.7% (586)	11.2% (614)	12.3% (423)	10.8% (255)
≥3	4.7% (270)	4.3% (213)	3.8% (210)	3.5% (121)	3.9% (92)

^a^ Time since the most recent evidence of a start of an episode; with episodes beginning after 2 months before the SARS‐CoV‐2 diagnosis considered as ongoing.

^b^ Hypertension, diabetes, chronic obstructive pulmonary disease, chronic kidney disease and tuberculosis (at any time).

**Table 4:** HIV characteristics and vaccination status for SARS‐CoV‐2 infections among adults with HIV in the Western Cape, stratified by wave (percentages and counts).

Characteristic	Wave 1 *n* = 5783	Wave 2 *n* = 5001	Wave 3 *n* = 5478	Wave 4 *n* = 3430	Inter‐wave *n* = 2361
**CD4 count (cells/μl)**					
≥ 500	11.6 (670)	13.7 (684)	13.3 (729)	12.7 (437)	10.8 (255)
200–499	13.4 (775)	12.8 (642)	14.3 (783)	12.4 (426)	15.2 (360)
50–199	5.2 (301)	6.0 (300)	7.4 (407)	10.2 (349)	9.3 (219)
0–49	2.4 (140)	2.6 (132)	2.8 (152)	6.1 (210)	5.9 (139)
Unknown	67.4 (3897)	64.8 (3243)	62.2 (3407)	58.5 (2008)	58.9 (1392)
**Viral load** ^a^ **(copies/ml)**					
0–999	74.7 (3970)	72.7 (3301)	69.7 (3449)	59.7 (1833)	64.4 (1360)
≥ 1000	6.6 (352)	8.9 (405)	10.8 (532)	13.3 (407)	14.5 (307)
Unknown	18.7 (991)	18.4 (837)	19.5 (966)	27.0 (830)	21.0 (444)
**ART (years since most recent collection)**					
≥ 2	49.6 (2868)	41.2 (2061)	45.9 (2517)	49.9 (1713)	41.7 (987)
< 2	42.3 (2445)	49.6 (2482)	44.4 (2430)	39.6 (1357)	47.5 (1124)
No ART	8.1 (470)	9.2 (458)	9.7 (531)	10.5 (360)	10.7 (254)
**HIV evidence (years since first)**					
<1	8.0 (464)	6.9 (347)	6.3 (346)	8.8 (303)	10.4 (246)
1–5	31.7 (1835)	32.0 (1602)	29.3 (1607)	29.3 (1006)	32.8 (776)
5–10	34.9 (2016)	34.8 (1738)	37.0 (2026)	35.7 (1224)	32.9 (777)
≥ 10	25.4 (1468)	26.3 (1314)	27.4 (1499)	26.2 (897)	23.9 (566)
**Vaccination status** ^b^					
None	100.0 (5783)	100.0 (5001)	88.8 (4866)	54.4 (1866)	94.9 (2245)
Early	−	−	5.5 (298)	1.7 (57)	1.0 (24)
Janssen—one vaccine	−	−	3.8 (207)	16.8 (577)	1.5 (35)
Pfizer—one vaccine	−	−	1.7 (91)	5.1 (174)	1.1 (26)
Janssen—two vaccines	−	−	0.0 (0)	3.9 (133)	0.2 (4)
Pfizer—two vaccines	−	−	0.3 (16)	18.2 (623)	1.3 (31)

Abbreviation: ART, antiretroviral therapy.

^a^ Among those with evidence of ART at any time.

^b^ The highest level of protection from among six categories: no vaccine received (“None”); <28 days since a first vaccine dose (“Early”); ≥28 days since a first dose of a vaccine, by type (“1 vaccine”); or ≥14 days since a second dose, by type (“2 vaccines”).

### 3.1 Associations with mortality: 15–39‐years‐old

**Figure 2:**
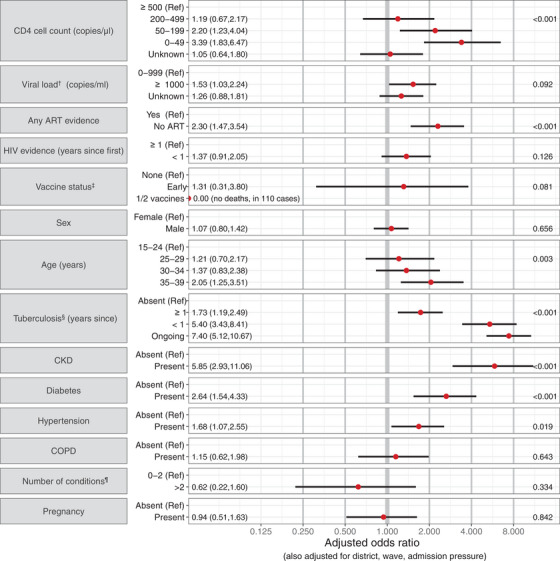
Adjusted odds ratios (and 95% CIs) describing the association of mortality with a person's HIV characteristics, vaccination, demographic characteristics and co‐existing conditions, for 15–39‐year‐old adults with HIV and SARS‐CoV‐2 infections in the Western Cape. *p*‐Values are reported on the right. **^†^** Among those with evidence of ART at any time. **^‡^** The highest level of protection from among: No vaccine received (“None”); <28 days since a first vaccine dose (“Early”); ≥28 days since (at least) a first dose of a vaccine (1 or 2 Pfizer or Janssen vaccines not distinguished due to small samples). ^§^ Time since the most recent evidence of a start of an episode; with episodes beginning after 2 months before the SARS‐CoV‐2 diagnosis considered as ongoing. ^¶^ Hypertension, diabetes, chronic obstructive pulmonary disease, chronic kidney disease and tuberculosis (at any time). Abbreviations: ART, antiretroviral therapy; CI, confidence interval; CKD, chronic kidney disease; COPD, chronic obstructive pulmonary disease.

Estimated from 9146 first diagnosed infections, the mortality risk was 2.9% (95% CI: 2.6,3.2%). Higher mortality was associated with being older, and with measures indicating worse HIV control (Figure 2), that is lower recent CD4 (aOR [95% CI]: 3.39 [1.83,6.47] for 0–49 cells/μL and 2.20 [1.23,4.04] for 50–199 cells/μL, each vs. ≥500); no evidence of ART collection (2.30 [1.47,3.54]); among those on ART, high or unknown recent VL (1.53 [1.03,2.24] for ≥1000 copies/ml and 1.26 [0.88,1.81] for unknown, vs. <1000); and recent first HIV evidence (1.37 [0.91,2.05] for evidence <1 year ago). For other comorbidities, in order of decreasing magnitudes of associations, those with ongoing tuberculosis, CKD, tuberculosis diagnosed <1 year ago, diabetes, tuberculosis diagnosed ≥1 year ago or hypertension experienced 2–8 times higher odds of mortality, though there may be an attenuation of the individual condition effects in those with multiple conditions. There were no deaths among the 110 infections that occurred in people who had received ≥1 vaccine. Higher admission pressure and later waves were associated with higher mortality (see SDC B for all univariable and multivariable results).

### 3.2 Associations with mortality: ≥40 years old

**Figure 3:**
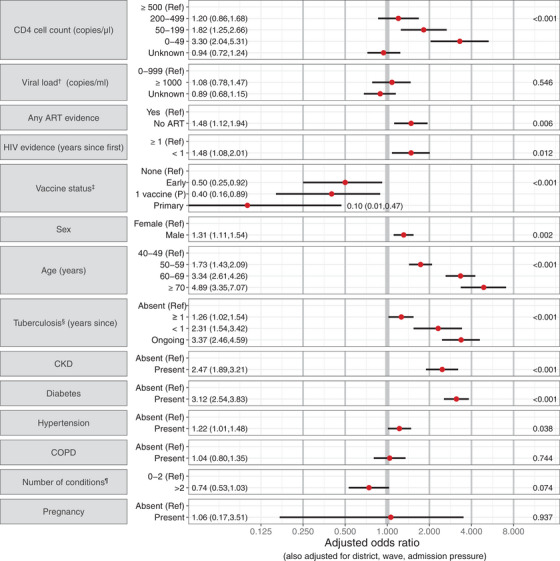
Adjusted odds ratios describing the association of mortality with a person's HIV characteristics, vaccination, demographic characteristics and co‐existing conditions, for adults with HIV and SARS‐CoV‐2 infections aged ≥ 40 years in the Western Cape. *p*‐Values are reported on the right. **^†^** Among those with evidence of ART at any time. **^‡^** The highest level of protection from among: No vaccine received (“None”); <28 days since a first vaccine dose (“Early”); ≥28 days since one dose of a Pfizer vaccine (“1 vaccine (P)”); ≥28 days since one Janssen vaccine, possibly with a second vaccine too, or ≥14 days since a second Pfizer vaccine (combined into the “primary” series due to the small sample). ^§^ Time since the most recent evidence of a start of an episode; with episodes beginning after 2 months before the SARS‐CoV‐2 diagnosis considered as ongoing. ^¶^ Hypertension, diabetes, chronic obstructive pulmonary disease, chronic kidney disease and tuberculosis (at any time). Abbreviations: ART, antiretroviral therapy; CKD, chronic kidney disease; COPD, chronic obstructive pulmonary disease.

Estimated from 8675 first diagnosed infections, the mortality risk was 8.6% (95% CI: 8.1,9.2%). Older age was most strongly associated with higher mortality with a moderate increase in mortality in men (Figure 3). As in the younger age group, most measures of poor HIV control were associated with higher mortality: lower recent CD4 (aOR [95% CI]: 3.30 [2.04,5.31] for 0–49 cells/μL and 1.82 [1.25,2.66] for 50–199, vs. ≥500), no ART (1.48 [1.12,1.94]) and having first HIV evidence within the last year (1.48 [1.08,2.01]); though there was no evidence suggesting an association with VL in those on ART. While comorbidities were also strongly associated with mortality, the associations were attenuated compared to those in 15–39‐year‐olds. Vaccination was strongly protective (0.10 [0.01; 0.47] for the primary series and 0.40 [0.16; 0.89] for one Pfizer vaccine dose, vs. no vaccine).

### 3.3 Secondary analyses

The associations of mortality with CD4 were similar (younger age group) or slightly dampened (older age group) when using CD4 nadir, while CD4 at ART initiation offered the least predictive power (see SDC C). Overall findings remained similar when including wave 4 infections (see SDC D).

## 4 DISCUSSION

Among more than 17,000 first diagnosed SARS‐CoV‐2 infections in adult PWH during the ancestral, Beta and Delta variant waves, mortality was strongly associated with suboptimal HIV control (CD4 cell count <200 cells/μl, no evidence of starting ART, recent first HIV evidence, and, in those aged <40 years, VL ≥1000 copies/ml despite ART). The prevalence of these risk factors increased during COVID‐19 waves 1 to 4. Mortality was also associated with the well‐established risk factors of older age, male sex (in those ≥40 years old) and comorbidities, including tuberculosis, which were present in half of those with SARS‐CoV‐2 infections. Vaccination was strongly protective against death.

To our knowledge, this is the largest study of SARS‐CoV‐2 infections, including non‐hospitalized PWH, and one of few studies from sub‐Saharan Africa where most PWH live and a greater proportion may be immunosuppressed and/or not on suppressive ART, allowing us to robustly assess their associations with mortality [1, 7, 8]. Our findings that low current CD4 cell count and HIV viraemia are associated with COVID‐19 mortality concur with several studies [4, 7, 8, 19–24]. We also found strong associations of mortality with nadir CD4 as previously reported from a large US cohort [25], recent HIV diagnosis, and previous and especially recent/current tuberculosis co‐infection [7]. These characteristics all suggest previous delayed HIV diagnosis and/or untreated or advanced HIV, highlighting the importance of strengthening HIV and tuberculosis care programmes to reduce vulnerability to SARS‐CoV‐2 and other infections. While most people in our cohort were known to be living with HIV for several years, 2% had the first evidence within 3 weeks of the SARS‐CoV‐2 diagnosis, suggesting both diagnoses may have occurred in the same health encounter with possible previous missed opportunities for HIV diagnosis and treatment.

The wave pattern observed in our study population (a relatively large first wave) mirrored that in the lower socio‐economic status subdistricts in the WC [26, 27], consistent with the burden of HIV in SA being disproportionately higher among the poor. Concerningly, we found an increasing and high proportion of patients with suboptimal HIV control in the later SARS‐CoV‐2 waves, possibly reflecting gaps in HIV care earlier in the pandemic [28, 29, 30, 31].

The importance of comorbidities in driving mortality, especially in younger PWH, has been demonstrated in several studies [1, 5–8, 32]. The high prevalence of comorbidities is notable; however, this may be impacted by testing guidelines during COVID‐19 waves restricting testing to older people or those with comorbidities [26]. These restrictions may also have biased towards PWH with poorer disease control, since younger virologically suppressed PWH were not eligible for testing during earlier COVID‐19 waves. Nonetheless, the high proportion of SARS‐CoV‐2 infections with previous/ongoing tuberculosis and associated increased risk of mortality confirms our earlier findings and it remains difficult to determine the extent of exacerbation of tuberculosis by SARS‐CoV‐2 infection and vice versa [3, 33].

Our findings have important implications for reducing poor SARS‐CoV‐2 outcomes in PWH. First, it remains a public health priority to ensure all PWH are on suppressive ART, as suboptimal HIV control is a key correlate of SARS‐CoV‐2 mortality and a determinant of prolonged infection with the risk of accumulation of mutations [13, 14]. We need to identify optimal strategies to reach PWH in the context of a pandemic [31, 34]. Second, we need to determine and implement the most effective COVID‐19 vaccination strategies for PWH, as primary vaccination was strongly protective against death in PWH with SARS‐CoV‐2 infection [10]. The proportion of persons who had received their primary vaccines increased from wave 3 (4.1%) to wave 4 (38.9%), likely due to several factors, including primary vaccines only being available to all adults towards the end of wave 3, the risk of vaccine breakthrough increasing with time since vaccination and increasing mismatch between the vaccine and circulating SARS‐CoV‐2 strain. Third, comorbidity diagnosis and management in PWH should be optimized, with a focus on tuberculosis diagnosis and treatment which has been severely impacted by COVID‐19 [35]. Finally, PWH with SARS‐CoV‐2 and suboptimal HIV control should be considered for anti‐SARS‐CoV‐2 therapy if available, in addition to using the COVID‐19 episode as an opportunity to optimize HIV management.

While strengths of our study include the large size and ability to link to pre‐existing HIV care and comorbidity data with ascertainment of ART, VL and CD4 cell count measures not reliant on patient recall, it also has several limitations. Routine public sector data may not include ART and other medications dispensed in the private sector, and comorbidities are likely under‐ascertained due to reliance on algorithmic inference based on public sector laboratory tests, ICD‐10 coding and treatments. We were unable to adjust for comorbidities and behaviours not algorithmically inferred. Recent CD4 cell counts were not available for >60% of PWH and those with measurements may be biased towards being unwell or starting/re‐starting ART as CD4 cell count measurement is not done routinely in those stable on ART for >1 year who are virologically suppressed. The large proportion of patients on ART with no documented ART collection in the last 2 years could be an over‐estimate as not all facilities used electronic dispensing. Longer scripting intervals and altered patterns of movement and medication collection during the pandemic may also have impacted the completeness of ART collection data. Nonetheless, a high proportion of those ever on ART had VL measured (81%) and, of those, 89% were virally suppressed, suggesting that most were receiving suppressive ART.

Interpretation of our results is restricted to those with diagnosed SARS‐CoV‐2 infections, which represent only a small fraction of all infections [36]. We, therefore, also did not assess the impact of prior SARS‐CoV‐2 infection on COVID‐19 outcomes. We restricted our analysis to first‐diagnosed infections, with a low risk of reinfection during our analysis period before the Omicron variant emerged. Compared to the population of all people with SARS‐CoV‐2 infection, our population will over‐represent those with better testing coverage, especially during waves when public sector testing was restricted, which includes older patients, pregnant people and those with more severe HIV, other comorbidities or severe COVID‐19 disease. Increased testing in those with severe COVID‐19 disease also likely attenuated the associations of mortality with HIV control indicators [37, 38, 39]. While we could not distinguish between deaths due to COVID‐19 per se, and those where the SARS‐CoV‐2 diagnosis was incidental with an alternative primary cause of death, to mitigate against this, we restricted our primary analysis to the first three waves when most patients dying with SARS‐COV‐2 had COVID‐19 pneumonia [18]. However, similar associations of mortality with HIV disease severity characteristics were found when including wave 4, and it is reasonable to expect that these characteristics will remain important risk factors due to future variants. Lack of specificity of the COVID‐19 death outcome and other diagnoses such as *Pneumocystis jiroveci* pneumonia (PJP) in these patients means that some of the association between low CD4 cell count and mortality in patients with COVID‐19 may be from pathology due to PJP rather than COVID‐19 itself.

## 5 CONCLUSIONS

In our analysis of routine health service data of PWH with SARS‐CoV‐2 infections, markers of suboptimal HIV management increased in later waves and are modifiable risk factors for poor COVID‐19 outcomes in addition to tuberculosis and other comorbidities, and should be considered when prioritizing patients for anti‐SARS‐CoV‐2 therapy. Maximizing efforts to ensure that PWH are on suppressive ART with optimal comorbidity diagnosis and management as well as COVID‐19 vaccination should be public health priorities for preventing both poor COVID‐19 and HIV outcomes.

## COMPETING INTERESTS

There are no competing interests.

## AUTHORS’ CONTRIBUTIONS

M‐AD, AB, NZ, RK and NT conceived the study. NZ prepared the data extracts. RK led the analysis of the data and produced manuscript outputs, with analysis contributions by NZ, ON, RO‐Y, TJ and SB, and critical reviews of the analysis outputs by M‐AD, AB, EM, VT and NT. RK, M‐AD and NZ drafted and finalized the manuscript. All authors reviewed the manuscript, provided inputs and approved the manuscript.

## FUNDING

We acknowledge funding for the Western Cape Provincial Health Data Centre from the Western Cape Department of Health, the US National Institutes for Health (R01 HD080465, U01 AI069924), the Bill and Melinda Gates Foundation (1164272, 1191327), the United States Agency for International Development (72067418CA00023), the European Union (101045989) and the Grand Challenges ICODA pilot initiative delivered by Health Data Research UK and funded by the Bill & Melinda Gates and Minderoo Foundations (INV‐017293). Funding was also received from Wellcome Trust (203135/Z/16/Z, 222574).

## DISCLAIMER

The funders had no role in the study design, data collection, data analysis, data interpretation or writing of this report. The opinions, findings and conclusions expressed in this manuscript reflect those of the authors alone.

## Supporting information

Supporting Information
Supporting information file: Supplemental Digital Content
PDF. Supplementary analysis outputs.
**Table A1**: Admission pressure, timing and location for SARS‐CoV‐2 infections among adults with HIV in the Western Cape, stratified by age group (percentages and counts).
**Table A2**: Alternative CD4 cell count measures for SARS‐CoV‐2 infections among adults with HIV in the Western Cape, stratified by age group (percentages and counts).
**Table B1**: Mortality rates, unadjusted and adjusted odds ratios (and 95% CIs) describing the association of mortality with a person's demographic characteristics, co‐existing conditions, vaccination status, HIV characteristics and time/location covariates, for 15–39‐year‐old adults with HIV and SARS‐CoV‐2 infections in the Western Cape.
**Table B2**: Mortality rates, unadjusted and adjusted odds ratios (and 95% CIs) describing the association of mortality with a person's demographic characteristics, co‐existing conditions, vaccination status, HIV characteristics and time/location covariates, for adults with HIV and SARS‐CoV‐2 infections ≥ 40 years old in the Western Cape.
**Table C1**: Adjusted odds ratios (and 95% CIs) describing the association of mortality with a person's demographic characteristics, co‐existing conditions, vaccination status, HIV characteristics and time/location covariates, using alternative CD4 cell count measures for 15–39‐year‐old adults with HIV and SARS‐CoV‐2 infections in the Western Cape.
**Table C2**: Adjusted odds ratios (and 95% CIs) describing the association of mortality with a person's demographic characteristics, co‐existing conditions, vaccination status, HIV characteristics and time/location covariates, using alternative CD4 cell count measures, for adults with HIV and SARS‐CoV‐2 infections ≥ 40 years old in the Western Cape.
**Figure D1**: Adjusted odds ratios (and 95% CIs) describing the association of mortality with a person's HIV characteristics, vaccination, demographic characteristics, and co‐existing conditions, for 15–39‐year‐old adults with HIV and SARS‐CoV‐2 infections in the Western Cape using all infections until 10 March 2022. P‐values are reported on the right.
**Figure D2**: Adjusted odds ratios (and 95% CIs) describing the association of mortality with a person's HIV characteristics, vaccination, demographic characteristics, and co‐existing conditions, for adults with HIV and SARS‐CoV‐2 infections aged ≥ 40 years in the Western Cape using all infections until 10 March 2022. P‐values are reported on the right.

## Data Availability

The data are not publicly available due to privacy or ethical restrictions. The data that support the findings of this study can be requested from the Western Cape Provincial Health Data Centre (WCPHDC) [https://www.westerncape.gov.za/general‐publication/provincial‐health‐data‐centre]; restrictions apply to the availability of these data.
