# Supplementary material for: COVID‐19 among adults living with HIV: correlates of mortality among public sector healthcare users in Western Cape, South Africa
Source: J Int AIDS Soc. 2023 Jun 20;26(6):e26104. doi: 10.1002/jia2.26104 (PMC10281639; doi:10.1002/jia2.26104)
Supplement: Supplementary file 1 — Supporting Information Supporting information file: Supplemental Digital Content PDF. Supplementary analysis outputs. Table A1: Admission pressure, timing and location for SARS‐CoV‐2 infections among adults with HIV in the Western Cape, stratified by age group (percentages and counts). Table A2: Alternative CD4 cell count measures for SARS‐CoV‐2 infections among adults with HIV in the Western Cape, stratified by age group (percentages and counts). Table B1: Mortality rates, unadjusted and adjusted odds ratios (and 95% CIs) describing the association of mortality with a person's demographic characteristics, co‐existing conditions, vaccination status, HIV characteristics and time/location covariates, for 15–39‐year‐old adults with HIV and SARS‐CoV‐2 infections in the Western Cape. Table B2: Mortality rates, unadjusted and adjusted odds ratios (and 95% CIs) describing the association of mortality with a person's demographic characteristics, co‐existing conditions, vaccination status, HIV characteristics and time/location covariates, for adults with HIV and SARS‐CoV‐2 infections ≥ 40 years old in the Western Cape. Table C1: Adjusted odds ratios (and 95% CIs) describing the association of mortality with a person's demographic characteristics, co‐existing conditions, vaccination status, HIV characteristics and time/location covariates, using alternative CD4 cell count measures for 15–39‐year‐old adults with HIV and SARS‐CoV‐2 infections in the Western Cape. Table C2: Adjusted odds ratios (and 95% CIs) describing the association of mortality with a person's demographic characteristics, co‐existing conditions, vaccination status, HIV characteristics and time/location covariates, using alternative CD4 cell count measures, for adults with HIV and SARS‐CoV‐2 infections ≥ 40 years old in the Western Cape. Figure D1: Adjusted odds ratios (and 95% CIs) describing the association of mortality with a person's HIV characteristics, vaccination, demographic characteristics, and co‐exi [file JIA2-26-e26104-s001.pdf]

Supplemental Digital Content

Additional analysis outputs for:

COVID-19 among adults living with HIV: Correlates of mortality among public sector healthcare users in Western Cape, South Africa

Reshma Kassanjee[1§], Mary-Ann Davies[1,2], Olina Ngwenya[3], Richard Osei-Yeboah [4], Theuns Jacobs[1,2], Erna Morden[1,2], Venessa Timmerman[2], Stefan Britz[5], Marc Mendelson[6], Jantjie Taljaard[7], Julien Riou[8], Andrew Boulle[1,2], Nicki Tiffin[2,9], Nesbert Zinyakatira[1,2]

1 School of Public Health, University of Cape Town, Cape Town, South Africa
2 Western Cape Government: Health and Wellness, Cape Town, South Africa
3 Wellcome Centre for Infectious Disease Research in Africa, Institute of Infectious Diseases and Molecular Medicine, University of Cape Town, Cape Town, South Africa
4 Division of Computational Biology, Integrative Biomedical Sciences Department, Faculty of Health Sciences, University of Cape Town, Cape Town, South Africa
5 Department of Statistical Sciences, University of Cape Town, Cape Town, South Africa
6 Division of Infectious Diseases and HIV Medicine, Department of Medicine, Groote Schuur Hospital, University of Cape Town, South Africa
7 Division of Infectious Diseases, Department of Medicine, Tygerberg Hospital, Stellenbosch University, South Africa
8 Institute of Social and Preventive Medicine, University of Bern, Bern, Switzerland
9 University of the Western Cape, Cape Town, South Africa

§ Corresponding author: Reshma Kassanjee
Reshma.Kassanjee@uct.ac.za

A: Additional descriptions of SARS-CoV-2 infections	2
B: Unadjusted and adjusted odds ratios for all model terms	3
C: Adjusted odds ratios using alternative CD4 cell counts measures	7
D: Adjusted odds ratios for models including SARS-CoV-2 infections diagnosed during Wave 4	11

Supplemental Digital Content A: Additional descriptions of SARS-CoV-2 infections

Table A1: Admission pressure, timing and location for SARS-CoV-2 infections among adults with HIV in the Western Cape, stratified by age group (percentages and counts)

Characteristic	Cases until end of Wave 3 Ages 15-39 years n = 9,321	Ages ≥40 years n = 8,799	All ages n = 18,120	All cases (including Wave 4) All ages n = 22,057
**Admission pressure[1] (%)**				
<25	28.7 (2,674)	23.5 (2,070)	26.2 (4,744)	29.2 (6,432)
≥25 and <50	34.1 (3,181)	36.5 (3,208)	35.3 (6,389)	37.8 (8,346)
≥50	37.2 (3,466)	40.0 (3,521)	38.6 (6,987)	33.0 (7,279)
**Wave**				
Wave 1	32.7 (3,052)	31.0 (2,731)	31.9 (5,783)	26.2 (5,783)
Wave 2	26.5 (2,469)	28.8 (2,532)	27.6 (5,001)	22.7 (5,001)
Wave 3	29.3 (2,731)	31.2 (2,747)	30.2 (5,478)	24.8 (5,478)
Wave 4	-	-	-	15.6 (3,430)
Inter-waves	11.5 (1,069)	9.0 (789)	10.3 (1,858)	10.7 (2,365)
**District**				
Cape Winelands	9.8 (915)	10.8 (950)	10.3 (1,865)	10.2 (2,253)
Central Karoo	1.1 (106)	1.4 (124)	1.3 (230)	1.3 (277)
City of Cape Town	61.1 (5,698)	62.7 (5,518)	61.9 (11,216)	62.1 (13,707)
Garden Route	18.0 (1,677)	16.1 (1,416)	17.1 (3,093)	17.2 (3,792)
Overberg	5.2 (489)	4.3 (379)	4.8 (868)	4.6 (1,009)
West Coast	4.7 (436)	4.7 (412)	4.7 (848)	4.6 (1,019)

[1] The week's total number of COVID-19 admissions relative to the maximum weekly COVID-19 admissions for that district (as a percent).

Table A2: Alternative CD4 cell count measures for SARS-CoV-2 infections among adults with HIV in the Western Cape, stratified by age group (percentages and counts)

CD4 count (cells/μl) measure	Cases until end of Wave 3 Ages 15-39 years n = 9,321	Ages ≥40 years n = 8,799	All ages n = 18,120	All cases (including Wave 4) All ages n = 22,057
**Recent**				
≥ 500	14.0 (1,304)	11.3 (991)	12.7 (2,295)	12.6 (2,775)
200-499	15.7 (1,465)	11.5 (1,014)	13.7 (2,479)	13.5 (2,986)
50-199	7.0 (648)	5.8 (511)	6.4 (1,159)	7.1 (1,576)
0-49	3.6 (333)	2.2 (191)	2.9 (524)	3.5 (773)
Unknown	59.8 (5,571)	69.2 (6,092)	64.4 (11,663)	63.2 (13,947)
**Nadir**				
≥ 500	15.3 (1,424)	12.1 (1,067)	13.7 (2,491)	13.4 (2,965)
200-499	44.1 (4,109)	39.2 (3,446)	41.7 (7,555)	40.7 (8,986)
50-199	21.3 (1,987)	28.7 (2,526)	24.9 (4,513)	25.1 (5,535)
0-49	8.0 (743)	10.1 (886)	9.0 (1,629)	9.6 (2,112)
Unknown	11.4 (1,058)	9.9 (874)	10.7 (1,932)	11.1 (2,459)
**At ART initiation**				
≥ 500	11.9 (1,105)	7.2 (631)	9.6 (1,736)	9.6 (2,111)
200-499	34.0 (3,166)	25.5 (2,245)	29.9 (5,411)	29.8 (6,572)
50-199	14.9 (1,387)	19.9 (1,752)	17.3 (3,139)	17.3 (3,813)
0-49	3.8 (358)	5.4 (477)	4.6 (835)	4.7 (1,027)
Unknown	35.5 (3,305)	42.0 (3,694)	38.6 (6,999)	38.7 (8,534)

Supplemental Digital Content B: Unadjusted and adjusted odds ratios for all model terms

Table B1: Mortality rates, unadjusted and adjusted odds ratios (and 95% CIs) describing the association of mortality with a person's demographic characteristics, co-existing conditions, vaccination status, HIV characteristics and time/location covariates, for 15–39-year-old adults with HIV and SARS-CoV-2 infections in the Western Cape

Variable	Category	n	Mortality risk (95% CI)	Univariable analysis: Unadjusted odds ratio (uOR)		Multivariable analysis: Adjusted odds ratio (aOR)	
				Estimate (95% CI)	p-value	Estimate (95% CI)	p-value
**Sex**	**Female (Ref)**	7114	2.52 (2.16,2.91)	-	<0.001		0.656
	**Male**	2032	4.13 (3.31,5.09)	1.67 (1.28,2.17)		1.07 (0.80,1.42)	
**Age group (years)**	**15-24 (Ref)**	884	2.26 (1.39,3.47)	-	0.001		0.003
	**25-29**	1973	2.13 (1.54,2.87)	0.94 (0.56,1.64)		1.21 (0.70,2.17)	
	**30-34**	3008	2.56 (2.03,3.19)	1.13 (0.70,1.92)		1.37 (0.83,2.38)	
	**35-39**	3281	3.78 (3.15,4.49)	1.70 (1.08,2.82)		2.05 (1.25,3.51)	
**Hypertension**	**Absent (Ref)**	8360	2.75 (2.41,3.12)	-	0.029		0.019
	**Present**	786	4.20 (2.91,5.85)	1.55 (1.05,2.22)		1.68 (1.07,2.55)	
**Diabetes**	**Absent (Ref)**	8789	2.74 (2.41,3.11)	-	0.001		<0.001
	**Present**	357	6.16 (3.90,9.18)	2.33 (1.45,3.57)		2.64 (1.54,4.33)	
**Chronic obstructive pulmonary disease**	**Absent (Ref)**	8776	2.83 (2.49,3.19)	-	0.19		0.643
	**Present**	370	4.05 (2.29,6.60)	1.45 (0.82,2.39)		1.15 (0.62,1.98)	
**Chronic kidney disease**	**Absent (Ref)**	9062	2.74 (2.41,3.09)	-	<0.001		<0.001
	**Present**	84	17.86 (10.35,27.74)	7.73 (4.20,13.32)		5.85 (2.93,11.06)	
**Tuberculosis[1] (years since)**	**Absent (Ref)**	6708	1.46 (1.19,1.78)	-	<0.001		<0.001
	**≥ 1**	1644	2.98 (2.21,3.92)	2.07 (1.45,2.92)		1.73 (1.19,2.49)	
	**< 1**	319	13.17 (9.66,17.38)	10.23 (6.93,14.87)		5.40 (3.43,8.41)	
	**Ongoing**	475	15.58 (12.44,19.16)	12.45 (9.03,17.09)		7.40 (5.12,10.67)	
**Number of conditions[2]**	**0-2 (Ref)**	9076	2.81 (2.48,3.17)	-	0.001		0.334
	**>2**	70	11.43 (5.07,21.28)	4.46 (1.96,8.88)		0.62 (0.22,1.60)	
**Pregnancy**	**Absent (Ref)**	8381	2.97 (2.62,3.36)	-	0.054		0.842
	**Present**	765	1.83 (1.00,3.05)	0.61 (0.34,1.01)		0.94 (0.51,1.63)	

Continued on next page

Table B1 continued

Variable	Category	n	Mortality risk (95% CI)	Univariable analysis: Unadjusted odds ratio (uOR)		Multivariable analysis: Adjusted odds ratio (aOR)	
				Estimate (95% CI)	p-value	Estimate (95% CI)	p-value
**Vaccination status[3]**	**None (Ref)**	8967	2.90 (2.56,3.27)		0.032		0.081
	**Early**	69	4.35 (0.91,12.18)	1.52 (0.37,4.13)		1.31 (0.31,3.80)	
	**1/2 vaccines**	110	0.00 (0.00,3.30)	0.00 (no deaths)		0.00 (0.00,0.29)	
**CD4 count (cells/µl)**	**≥ 500 (Ref)**	1280	1.48 (0.90,2.31)	-	<0.001		<0.001
	**200-499**	1441	2.50 (1.76,3.44)	1.70 (0.98,3.04)		1.19 (0.67,2.17)	
	**50-199**	631	7.77 (5.80,10.14)	5.59 (3.32,9.80)		2.20 (1.23,4.04)	
	**0-49**	318	14.78 (11.07,19.17)	11.51 (6.76,20.38)		3.39 (1.83,6.47)	
	**Unknown**	5476	2.05 (1.69,2.46)	1.39 (0.87,2.33)		1.05 (0.64,1.80)	
**Viral load[4] (copies/ml)**	**0-999 (Ref)**	5546	1.77 (1.44,2.15)	-	<0.001		0.092
	**≥ 1000**	892	7.06 (5.47,8.95)	4.22 (3.04,5.83)		1.53 (1.03,2.24)	
	**Unknown**	1896	3.01 (2.28,3.88)	1.72 (1.23,2.39)		1.26 (0.88,1.81)	
**ART collection**	**Yes**	8334	2.62 (2.28,2.98)	-	<0.001		<0.001
	**No ART**	812	5.54 (4.07,7.35)	2.18 (1.55,3.01)		2.30 (1.47,3.54)	
**HIV (years since first evidence)**	**≥ 1 (Ref)**	8322	2.66 (2.32,3.02)	-	<0.001		0.126
	**< 1**	824	5.10 (3.70,6.83)	1.97 (1.39,2.73)		1.37 (0.91,2.05)	
**Admission pressure[5] (%)**	**<25 (Ref)**	2630	2.78 (2.18,3.48)	-	0.3		0.036
	**≥25 and <50**	3147	3.24 (2.65,3.92)	1.17 (0.87,1.60)		1.65 (1.11,2.49)	
	**≥50**	3369	2.61 (2.10,3.21)	0.94 (0.69,1.29)		1.24 (0.79,2.00)	
**Wave**	**Wave 1 (Ref)**	3052	2.23 (1.73,2.82)	-	0.008		0.022
	**Wave 2**	2415	2.61 (2.01,3.33)	1.18 (0.83,1.66)		1.29 (0.82,2.01)	
	**Wave 3**	2636	3.57 (2.89,4.35)	1.62 (1.18,2.23)		1.68 (1.13,2.50)	
	**Inter-waves**	1043	3.64 (2.59,4.97)	1.66 (1.10,2.47)		1.83 (1.08,3.11)	
**District**	**City of Cape Town (Ref)**	5599	2.82 (2.40,3.29)	-	0.209		0.136
	**Cape Winelands**	907	4.08 (2.89,5.58)	1.46 (1.00,2.09)		1.70 (1.12,2.52)	
	**Central Karoo**	104	1.92 (0.23,6.77)	0.68 (0.11,2.16)		0.50 (0.08,1.75)	
	**Garden Route**	1623	2.40 (1.71,3.27)	0.85 (0.59,1.20)		0.95 (0.64,1.37)	
	**Overberg**	483	3.52 (2.06,5.58)	1.26 (0.73,2.03)		1.37 (0.76,2.32)	
	**West Coast**	430	2.33 (1.12,4.24)	0.82 (0.40,1.49)		0.94 (0.45,1.75)	

[1] Time since the most recent evidence of a start of an episode; with episodes beginning after 2 months before the SARS-CoV-2 diagnosis considered as ongoing. [2] Hypertension, diabetes, chronic obstructive pulmonary disease, chronic kidney disease, tuberculosis (at any time). [3] The highest level of protection from among: No vaccine received ('None'); <28 days since a first vaccine dose ('Early'); ≥28 days since (at least) a first dose of a vaccine (1 or 2 Pfizer or Janssen vaccines not distinguished due to small samples). [4] Among those with evidence of ART at any time. [5] The week's total number of COVID-19 admissions relative to the maximum weekly COVID-19 admissions for that district (as a percent).
ART: Antiretroviral therapy. CI: Confidence interval.

Table B2: Mortality rates, unadjusted and adjusted odds ratios (and 95% CIs) describing the association of mortality with a person's demographic characteristics, co-existing conditions, vaccination status, HIV characteristics and time/location covariates, for adults with HIV and SARS-CoV-2 infections ≥ 40 years old in the Western Cape

Variable	Category	n	Mortality risk (95% CI)	Univariable analysis: Unadjusted odds ratio (uOR)		Multivariable analysis: Adjusted odds ratio (aOR)	
				Estimate (95% CI)	p-value	Estimate (95% CI)	p-value
**Sex**	**Female (Ref)**	5529	7.23 (6.57,7.95)	-	<0.001		0.002
	**Male**	3146	11.09 (10.02,12.24)	1.60 (1.38,1.86)		1.31 (1.11,1.54)	
**Age group (years)**	**40-49 (Ref)**	5207	5.36 (4.76,6.00)	-	<0.001		<0.001
	**50-59**	2560	10.35 (9.20,11.60)	2.04 (1.71,2.43)		1.73 (1.43,2.09)	
	**60-69**	711	20.68 (17.75,23.84)	4.60 (3.70,5.72)		3.34 (2.61,4.26)	
	**≥ 70**	197	29.44 (23.18,36.34)	7.37 (5.27,10.20)		4.89 (3.35,7.07)	
**Hypertension**	**Absent (Ref)**	5796	6.82 (6.18,7.49)	-	<0.001		0.039
	**Present**	2879	12.30 (11.12,13.55)	1.92 (1.65,2.23)		1.22 (1.01,1.48)	
**Diabetes**	**Absent (Ref)**	7298	6.48 (5.93,7.07)	-	<0.001		<0.001
	**Present**	1377	20.04 (17.96,22.26)	3.62 (3.08,4.25)		3.12 (2.54,3.83)	
**Chronic obstructive pulmonary disease**	**Absent (Ref)**	7782	8.30 (7.70,8.94)	-	0.002		0.744
	**Present**	893	11.53 (9.51,13.81)	1.44 (1.15,1.79)		1.04 (0.80,1.35)	
**Chronic kidney disease**	**Absent (Ref)**	8098	7.42 (6.86,8.01)	-	<0.001		<0.001
	**Present**	577	25.65 (22.13,29.42)	4.30 (3.50,5.27)		2.47 (1.89,3.21)	
**Tuberculosis[1] (years since)**	**Absent (Ref)**	5799	7.23 (6.57,7.92)	-	<0.001		<0.001
	**≥ 1**	2284	8.89 (7.75,10.13)	1.25 (1.05,1.49)		1.26 (1.02,1.54)	
	**< 1**	228	18.86 (14.00,24.55)	2.98 (2.09,4.18)		2.31 (1.54,3.42)	
	**Ongoing**	364	23.08 (18.85,27.75)	3.85 (2.95,4.99)		3.37 (2.46,4.59)	
**Number of conditions[2]**	**0-2 (Ref)**	7994	7.36 (6.79,7.95)	-	<0.001		0.074
	**>2**	681	23.64 (20.50,27.02)	3.90 (3.20,4.73)		0.74 (0.53,1.03)	
**Pregnancy**	**Absent (Ref)**	8625	8.66 (8.08,9.27)	-	0.195		0.937
	**Present**	50	4.00 (0.49,13.71)	0.44 (0.07,1.42)		1.06 (0.17,3.51)	

Continued on next page

Table B2 continued

Variable	Category	n	Mortality risk (95% CI)	Univariable analysis: Unadjusted odds ratio (uOR)		Multivariable analysis: Adjusted odds ratio (aOR)	
				Estimate (95% CI)	p-value	Estimate (95% CI)	p-value
**Vaccination status[3]**	**None (Ref)**	8275	8.82 (8.22,9.45)	-	0.001		<0.001
	**Early**	217	5.07 (2.56,8.89)	0.55 (0.28,0.97)		0.50 (0.25,0.92)	
	**Pfizer - 1 vaccine**	79	8.86 (3.64,17.41)	1.00 (0.42,2.04)		0.40 (0.16,0.89)	
	**Primary**	104	0.96 (0.02,5.24)	0.10 (0.01,0.45)		0.10 (0.01,0.47)	
**CD4 count (cells/μl)**	**≥ 500 (Ref)**	974	7.80 (6.20,9.67)	-	<0.001		<0.001
	**200-499**	998	10.12 (8.32,12.16)	1.33 (0.98,1.82)		1.20 (0.86,1.68)	
	**50-199**	502	14.74 (11.76,18.15)	2.04 (1.45,2.87)		1.82 (1.25,2.66)	
	**0-49**	184	24.46 (18.43,31.32)	3.83 (2.53,5.74)		3.30 (2.04,5.31)	
	**Unknown**	6017	7.53 (6.87,8.22)	0.96 (0.75,1.25)		0.94 (0.72,1.24)	
**Viral load[4] (copies/ml)**	**0-999 (Ref)**	6112	7.66 (7.00,8.35)	-	<0.001		0.546
	**≥ 1000**	570	12.63 (10.02,15.64)	1.74 (1.33,2.26)		1.08 (0.78,1.47)	
	**Unknown**	1183	7.27 (5.86,8.90)	0.95 (0.74,1.19)		0.89 (0.68,1.15)	
**ART collection**	**Yes**	7865	7.96 (7.37,8.58)	-	<0.001		0.006
	**No ART**	810	15.19 (12.78,17.84)	2.07 (1.67,2.54)		1.48 (1.12,1.94)	
**HIV (years since first evidence)**	**≥ 1 (Ref)**	8164	8.06 (7.48,8.67)	-	<0.001		0.012
	**< 1**	511	17.81 (14.59,21.41)	2.47 (1.93,3.13)		1.48 (1.08,2.01)	
**Admission pressure[5] (%)**	**<25 (Ref)**	2036	8.25 (7.09,9.53)	-	0.137		0.932
	**≥25 and <50**	3183	9.43 (8.43,10.49)	1.16 (0.95,1.41)		0.99 (0.78,1.27)	
	**≥50**	3456	8.13 (7.24,9.09)	0.98 (0.81,1.20)		0.95 (0.72,1.26)	
**Wave**	**Wave 1 (Ref)**	2731	9.34 (8.27,10.49)	-	0.286		0.104
	**Wave 2**	2505	8.58 (7.51,9.75)	0.91 (0.75,1.10)		0.96 (0.74,1.25)	
	**Wave 3**	2672	8.35 (7.32,9.46)	0.88 (0.73,1.07)		1.14 (0.89,1.46)	
	**Inter-waves**	767	7.30 (5.56,9.38)	0.76 (0.56,1.03)		0.74 (0.51,1.06)	
**District**	**City of Cape Town (Ref)**	5447	9.42 (8.66,10.22)	-	0.002		0.231
	**Cape Winelands**	941	9.03 (7.28,11.05)	0.96 (0.75,1.21)		0.97 (0.74,1.26)	
	**Central Karoo**	121	8.26 (4.03,14.67)	0.87 (0.42,1.58)		0.75 (0.35,1.47)	
	**Garden Route**	1388	6.56 (5.31,7.99)	0.67 (0.53,0.85)		0.82 (0.64,1.05)	
	**Overberg**	368	5.71 (3.57,8.59)	0.58 (0.36,0.89)		0.65 (0.39,1.03)	
	**West Coast**	410	7.07 (4.79,10.00)	0.73 (0.49,1.06)		0.77 (0.50,1.14)	

[1] Time since the most recent evidence of a start of an episode; with episodes beginning after 2 months before the SARS-CoV-2 diagnosis considered as ongoing. [2] Hypertension, diabetes, chronic obstructive pulmonary disease, chronic kidney disease, tuberculosis (at any time). [3] The highest level of protection from among: No vaccine received ('None'); <28 days since a first vaccine dose ('Early'); ≥28 days since 1 dose of a Pfizer vaccine ('1 vaccine (P)'); ≥28 days since 1 Janssen vaccine, possibly with a 2nd vaccine too, or ≥14 days since a 2nd Pfizer vaccine |(combined into the 'primary' series due to the small sample). [4] Among those with evidence of ART at any time. [5] The week's total number of COVID-19 admissions relative to the maximum weekly COVID-19 admissions for that district (as a percent).
ART: Antiretroviral therapy. CI: Confidence interval.

Supplemental Digital Content C: Adjusted odds ratios using alternative CD4 cell counts measures

Table C1: Adjusted odds ratios (and 95% CIs) describing the association of mortality with a person's demographic characteristics, co-existing conditions, vaccination status, HIV characteristics and time/location covariates, using alternative CD4 cell count measures for 15–39-year-old adults with HIV and SARS-CoV-2 infections in the Western Cape

		Adjusted odds ratio (aOR)		
**Variable**	**Category**	**Model 1: Recent CD4 (AIC[6]: 2051)**	**Model 2: CD4 nadir (AIC: 2043)**	**Model 3: CD4 at ART start (AIC: 2062)**
**Sex**	**Female (Ref)**			
	**Male**	1.07 (0.80,1.42)	1.04 (0.78,1.39)	1.09 (0.81,1.45)
**Age group (years)**	**15-24 (Ref)**			
	**25-29**	1.21 (0.70,2.17)	1.15 (0.66,2.06)	1.14 (0.66,2.03)
	**30-34**	1.37 (0.83,2.38)	1.30 (0.78,2.24)	1.28 (0.78,2.22)
	**35-39**	2.05 (1.25,3.51)	1.88 (1.15,3.21)	1.91 (1.17,3.27)
**Hypertension**	**Absent (Ref)**			
	**Present**	1.68 (1.07,2.55)	1.70 (1.08,2.59)	1.67 (1.06,2.53)
**Diabetes**	**Absent (Ref)**			
	**Present**	2.64 (1.54,4.33)	2.56 (1.49,4.20)	2.46 (1.44,4.01)
**Chronic obstructive pulmonary disease**	**Absent (Ref)**			
	**Present**	1.15 (0.62,1.98)	1.14 (0.62,1.97)	1.18 (0.64,2.03)
**Chronic kidney disease**	**Absent (Ref)**			
	**Present**	5.85 (2.93,11.06)	5.99 (3.01,11.31)	5.79 (2.90,10.95)
**Tuberculosis[1] (years since)**	**Absent (Ref)**			
	**≥ 1**	1.73 (1.19,2.49)	1.50 (1.01,2.20)	1.67 (1.14,2.42)
	**< 1**	5.40 (3.43,8.41)	5.81 (3.71,8.99)	7.48 (4.88,11.30)
	**Ongoing**	7.40 (5.12,10.67)	7.58 (5.24,10.96)	8.66 (6.06,12.36)
**Number of conditions[2]**	**0-2 (Ref)**			
	**>2**	0.62 (0.22,1.60)	0.61 (0.22,1.58)	0.64 (0.23,1.65)
**Pregnancy**	**Absent (Ref)**			
	**Present**	0.94 (0.51,1.63)	0.95 (0.51,1.64)	0.98 (0.53,1.68)
**Vaccination status[3]**	**None (Ref)**			
	**Early**	1.31 (0.31,3.80)	1.29 (0.30,3.71)	1.18 (0.28,3.46)
	**1/2 vaccines**	0.00 (0.00,0.29)	0.00 (0.00,0.00)	0.00 (0.00,0.00)
**CD4 count (cells/µl)**	**≥ 500 (Ref)**			
	**200-499**	1.19 (0.67,2.17)	1.08 (0.67,1.84)	0.90 (0.55,1.52)
	**50-199**	2.20 (1.23,4.04)	1.29 (0.76,2.25)	1.41 (0.84,2.43)
	**0-49**	3.39 (1.83,6.47)	3.36 (1.95,5.98)	1.60 (0.84,3.05)
	**Unknown**	1.05 (0.64,1.80)	1.16 (0.63,2.17)	0.63 (0.37,1.11)
**Viral load[4] (copies/ml)**	**0-999 (Ref)**			
	**≥ 1000**	1.53 (1.03,2.24)	1.68 (1.15,2.43)	2.11 (1.46,3.02)
	**Unknown**	1.26 (0.88,1.81)	1.35 (0.94,1.93)	1.43 (1.00,2.04)
**ART collection**	**Yes**			
	**No ART**	2.30 (1.47,3.54)	2.33 (1.45,3.66)	3.32 (1.97,5.62)
**HIV (years since first evidence)**	**≥ 1 (Ref)**			
	**< 1**	1.37 (0.91,2.05)	1.48 (0.96,2.24)	1.33 (0.88,1.99)

Continued on next page

Table C1 continued

Variable	Category	Adjusted odds ratio (aOR) Model 1: Recent CD4 (AIC[6]: 2051)	Model 2: CD4 nadir (AIC: 2043)	Model 3: CD4 at ART start (AIC: 2062)
**Admission pressure[5] (%)**	**<25 (Ref)**			
	**≥25 and <50**	1.65 (1.11,2.49)	1.68 (1.13,2.53)	1.66 (1.12,2.51)
	**≥50**	1.24 (0.79,2.00)	1.30 (0.82,2.09)	1.25 (0.80,2.01)
**Wave**	**Wave 1 (Ref)**			
	**Wave 2**	1.29 (0.82,2.01)	1.27 (0.81,1.99)	1.31 (0.83,2.04)
	**Wave 3**	1.68 (1.13,2.50)	1.71 (1.15,2.54)	1.66 (1.11,2.47)
	**Inter-waves**	1.83 (1.08,3.11)	1.81 (1.07,3.07)	1.88 (1.11,3.19)
**District**	**City of Cape Town (Ref)**			
	**Cape Winelands**	1.70 (1.12,2.52)	1.70 (1.12,2.54)	1.71 (1.12,2.54)
	**Central Karoo**	0.50 (0.08,1.75)	0.55 (0.09,1.88)	0.51 (0.08,1.77)
	**Garden Route**	0.95 (0.64,1.37)	0.94 (0.64,1.37)	0.96 (0.65,1.39)
	**Overberg**	1.37 (0.76,2.32)	1.38 (0.77,2.34)	1.31 (0.73,2.21)
	**West Coast**	0.94 (0.45,1.75)	0.93 (0.45,1.73)	0.96 (0.46,1.80)

[1] Time since the most recent evidence of a start of an episode; with episodes beginning after 2 months before the SARS-CoV-2 diagnosis considered as ongoing. [2] Hypertension, diabetes, chronic obstructive pulmonary disease, chronic kidney disease, tuberculosis (at any time). [3] The highest level of protection from among: No vaccine received ('None'); <28 days since a first vaccine dose ('Early'); ≥28 days since (at least) a first dose of a vaccine (1 or 2 Pfizer or Janssen vaccines not distinguished due to small samples). [4] Among those with evidence of ART at any time. [5] The week's total number of COVID-19 admissions relative to the maximum weekly COVID-19 admissions for that district (as a percent). [6] Akaike Information Criterion (AIC) provides relative measure of models' predictive powers; lower AIC values are more favourable.
ART: Antiretroviral therapy. CI: Confidence interval.

Table C2: Adjusted odds ratios (and 95% CIs) describing the association of mortality with a person's demographic characteristics, co-existing conditions, vaccination status, HIV characteristics and time/location covariates, using alternative CD4 cell count measures, for adults with HIV and SARS-CoV-2 infections ≥ 40 years old in the Western Cape

		Adjusted odds ratio (aOR)		
**Variable**	**Category**	**Model 1: Recent CD4 (AIC[6]: 4465)**	**Model 2: CD4 nadir (AIC: 4470)**	**Model 3: CD4 at ART start (AIC: 4489)**
**Sex**	**Female (Ref)**			
	**Male**	1.31 (1.11,1.54)	1.30 (1.10,1.53)	1.33 (1.13,1.57)
**Age group (years)**	**40-49 (Ref)**			
	**50-59**	1.73 (1.43,2.09)	1.71 (1.42,2.06)	1.67 (1.39,2.02)
	**60-69**	3.34 (2.61,4.26)	3.44 (2.68,4.39)	3.27 (2.56,4.17)
	**70+**	4.89 (3.35,7.07)	4.99 (3.41,7.24)	4.62 (3.16,6.68)
**Hypertension**	**Absent (Ref)**			
	**Present**	1.22 (1.01,1.48)	1.23 (1.02,1.49)	1.22 (1.01,1.48)
**Diabetes**	**Absent (Ref)**			
	**Present**	3.12 (2.54,3.83)	3.14 (2.56,3.86)	3.04 (2.48,3.73)
**Chronic obstructive pulmonary disease**	**Absent (Ref)**			
	**Present**	1.04 (0.80,1.35)	1.09 (0.84,1.41)	1.09 (0.84,1.41)
**Chronic kidney disease**	**Absent (Ref)**			
	**Present**	2.47 (1.89,3.21)	2.49 (1.91,3.23)	2.52 (1.93,3.27)
**Tuberculosis[1] (years since)**	**Absent (Ref)**			
	**12m+**	1.26 (1.02,1.54)	1.18 (0.96,1.45)	1.24 (1.01,1.52)
	**<12m**	2.31 (1.54,3.42)	2.68 (1.80,3.93)	2.93 (1.96,4.28)
	**Ongoing**	3.37 (2.46,4.59)	3.66 (2.69,4.96)	4.07 (2.99,5.49)
**Number of conditions[2]**	**0-2 (Ref)**			
	**>2**	0.74 (0.53,1.03)	0.75 (0.54,1.04)	0.75 (0.54,1.04)
**Pregnancy**	**Absent (Ref)**			
	**Present**	1.06 (0.17,3.51)	1.14 (0.18,3.74)	1.11 (0.18,3.65)
**Vaccination status[3]**	**None (Ref)**			
	**Early**	0.50 (0.25,0.92)	0.49 (0.24,0.90)	0.49 (0.24,0.90)
	**Pfizer - 1 vaccine**	0.40 (0.16,0.89)	0.41 (0.16,0.89)	0.41 (0.16,0.90)
	**Primary**	0.10 (0.01,0.47)	0.10 (0.01,0.48)	0.09 (0.01,0.44)
**CD4 count (cells/µl)**	**≥ 500 (Ref)**			
	**200-499**	1.20 (0.86,1.68)	1.37 (1.02,1.86)	1.07 (0.74,1.57)
	**50-199**	1.82 (1.25,2.66)	1.50 (1.11,2.07)	1.37 (0.95,2.01)
	**0-49**	3.30 (2.04,5.31)	2.36 (1.65,3.38)	2.11 (1.36,3.32)
	**Unknown**	0.94 (0.72,1.24)	0.79 (0.53,1.19)	1.21 (0.85,1.76)
**Viral load[4] (copies/ml)**	**0-999 (Ref)**			
	**≥ 1000**	1.08 (0.78,1.47)	1.34 (0.99,1.79)	1.45 (1.08,1.93)
	**Unknown**	0.89 (0.68,1.15)	1.03 (0.79,1.34)	0.94 (0.73,1.21)
**ART collection**	**Yes**			
	**No ART**	1.48 (1.12,1.94)	1.80 (1.32,2.43)	1.40 (1.03,1.88)
**HIV (years since first evidence)**	**≥ 1 (Ref)**			
	**< 1**	1.48 (1.08,2.01)	1.87 (1.35,2.57)	1.57 (1.15,2.14)

Continued on next page

Table C2 continued

Variable	Category	Adjusted odds ratio (aOR)		
		Model 1: Recent CD4 (AIC[6]: 4465)	Model 2: CD4 nadir (AIC: 4470)	Model 3: CD4 at ART start (AIC: 4489)
**Admission pressure[5] (%)**	**<25 (Ref)**			
	**≥25 and <50**	0.99 (0.78,1.27)	0.98 (0.77,1.26)	1.00 (0.78,1.28)
	**≥50**	0.95 (0.72,1.26)	0.95 (0.72,1.26)	0.95 (0.72,1.26)
**Wave**	**Wave 1 (Ref)**			
	**Wave 2**	0.96 (0.74,1.25)	0.97 (0.74,1.26)	0.98 (0.75,1.27)
	**Wave 3**	1.14 (0.89,1.46)	1.15 (0.90,1.48)	1.18 (0.91,1.51)
	**Inter-waves**	0.74 (0.51,1.06)	0.72 (0.50,1.04)	0.75 (0.52,1.09)
**District**	**City of Cape Town (Ref)**			
	**Cape Winelands**	0.97 (0.74,1.26)	1.01 (0.77,1.31)	1.00 (0.76,1.30)
	**Central Karoo**	0.75 (0.35,1.47)	0.81 (0.37,1.57)	0.79 (0.36,1.54)
	**Garden Route**	0.82 (0.64,1.05)	0.84 (0.65,1.07)	0.82 (0.64,1.05)
	**Overberg**	0.65 (0.39,1.03)	0.66 (0.40,1.05)	0.67 (0.40,1.06)
	**West Coast**	0.77 (0.50,1.14)	0.79 (0.52,1.18)	0.80 (0.52,1.18)

[1] Time since the most recent evidence of a start of an episode; with episodes beginning after 2 months before the SARS-CoV-2 diagnosis considered as ongoing. [2] Hypertension, diabetes, chronic obstructive pulmonary disease, chronic kidney disease, tuberculosis (at any time). [3] The highest level of protection from among: No vaccine received ('None'); <28 days since a first vaccine dose ('Early'); ≥28 days since 1 dose of a Pfizer vaccine ('1 vaccine (P)'); ≥28 days since 1 Janssen vaccine, possibly with a 2nd vaccine too, or ≥14 days since a 2nd Pfizer vaccine |(combined into the 'primary' series due to the small sample). [4] Among those with evidence of ART at any time. [5] The week's total number of COVID-19 admissions relative to the maximum weekly COVID-19 admissions for that district (as a percent). [6] Akaike Information Criterion (AIC) provides relative measure of models' predictive powers; lower AIC values are more favourable.
ART: Antiretroviral therapy. CI: Confidence interval.

Supplemental Digital Content D: Adjusted odds ratios for models including SARS-CoV-2 infections diagnosed during Wave 4

Figure D1: Adjusted odds ratios (and 95% CIs) describing the association of mortality with a person's HIV characteristics, vaccination, demographic characteristics, and co-existing conditions, for 15–39-year-old adults with HIV and SARS-CoV-2 infections in the Western Cape using all infections until 10 March 2022. P-values are reported on the right.

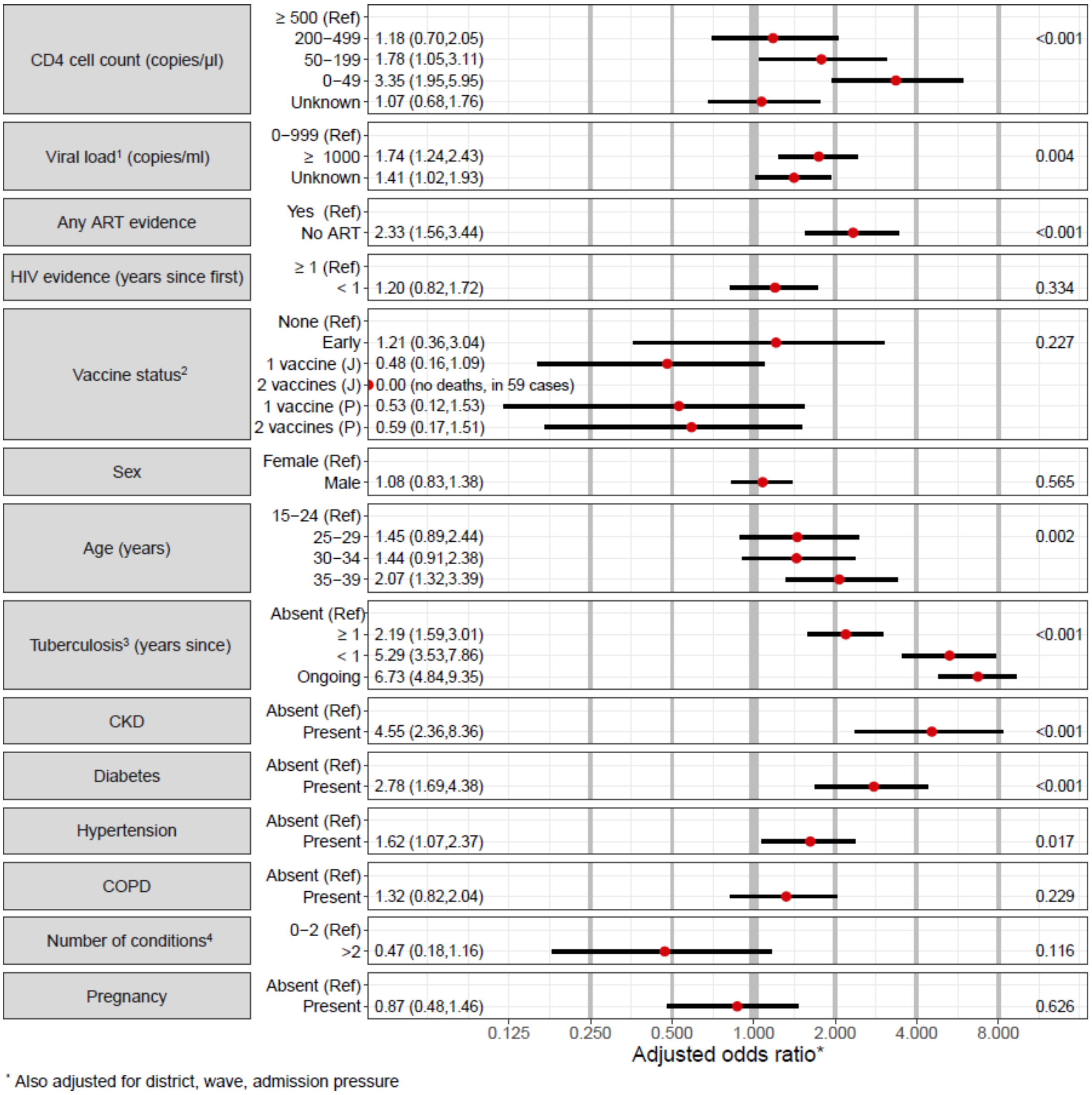

[1] Among those with evidence of ART at any time. [2] The highest level of protection from among six categories: No vaccine received ('None'); <28 days since a first vaccine dose ('Early'); ≥28 days since a first dose of a vaccine, by type ('1 vaccine'); or ≥14 days since a second dose, by type ('2 vaccines'). J: Janssen, P: Pfizer. [3] Time since the most recent evidence of a start of an episode; with episodes beginning after 2 months before the SARS-CoV-2 diagnosis considered as ongoing. [4] Hypertension, diabetes, chronic obstructive pulmonary disease, chronic kidney disease, tuberculosis (at any time).
ART: Antiretroviral therapy. CI: Confidence interval. CKD: Chronic kidney disease. COPD: Chronic obstructive pulmonary disease.

Figure D2: Adjusted odds ratios (and 95% CIs) describing the association of mortality with a person's HIV characteristics, vaccination, demographic characteristics, and co-existing conditions, for adults with HIV and SARS-CoV-2 infections aged ≥ 40 years in the Western Cape using all infections until 10 March 2022. P-values are reported on the right.

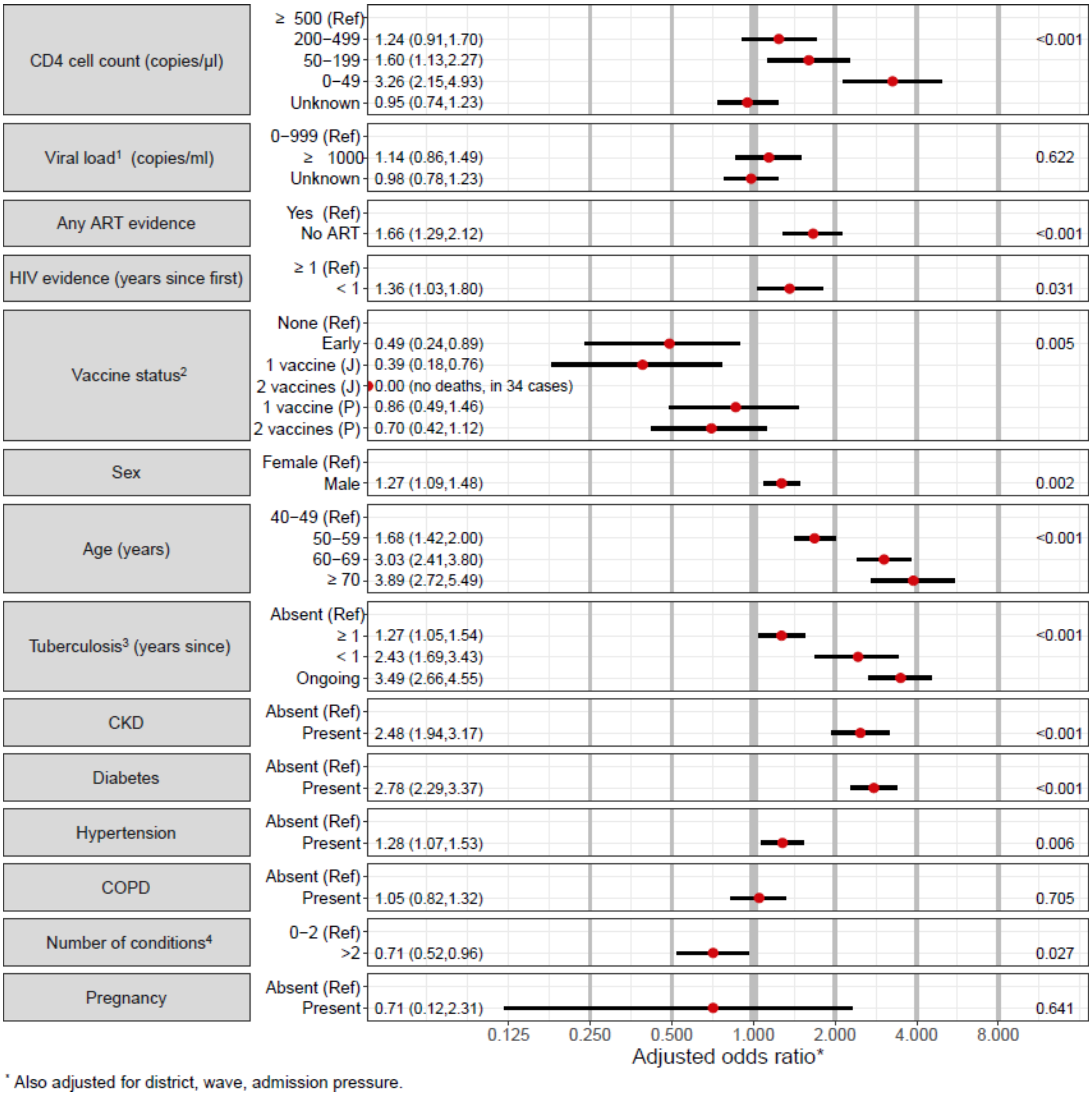

[1] Among those with evidence of ART at any time. [2] The highest level of protection from among six categories: No vaccine received ('None'); <28 days since a first vaccine dose ('Early'); ≥28 days since a first dose of a vaccine, by type ('1 vaccine'); or ≥14 days since a second dose, by type ('2 vaccines'). J: Janssen, P: Pfizer. [3] Time since the most recent evidence of a start of an episode; with episodes beginning after 2 months before the SARS-CoV-2 diagnosis considered as ongoing. [4] Hypertension, diabetes, chronic obstructive pulmonary disease, chronic kidney disease, tuberculosis (at any time).
ART: Antiretroviral therapy. CI: Confidence interval. CKD: Chronic kidney disease. COPD: Chronic obstructive pulmonary disease.
